# Supplementary material for: Scaffold-enabled high-resolution cryo-EM structure determination of RNA
Source: Nat Commun. 2025 Jan 21;16:880. doi: 10.1038/s41467-024-55699-5 (PMC11751092; doi:10.1038/s41467-024-55699-5)
Supplement: Supplementary file 1 — Supplementary Information [file 41467_2024_55699_MOESM1_ESM.pdf]

Scaffold-enabled high-resolution cryo-EM structure determination of RNA

Daniel B. Haack[1], Boris Rudolfs[1], Shouhong Jin[2], Alexandra Khitun[2], Kevin M. Weeks[2] and Navtej Toor[1*]

[1]Department of Chemistry and Biochemistry, University of California, San Diego, La Jolla, CA

[2]Department of Chemistry, University of North Carolina, Chapel Hill, NC

*Correspondence should be addressed to N.T. (ntoor@ucsd.edu)

This PDF file includes:

Supplementary Figure Legends
Figures S1 to S10
Tables S1

Supplementary Figure and Table Legends

Figure 1. Current state of cryo-EM on protein-free RNA samples. Unsharpened 3D reconstructions of a representative selection of protein-free RNAs from the RCSB Protein Data Bank are shown. The glycine riboswitch from V. cholera (pink, PDB-6WLU) is a 231-nt RNA and 3D reconstruction was determined to a reported resolution of 5.7 Å. A 3D reconstruction of a 171-nt tRNA-like structure from B. mosaic (purple, PDB-7SAM) was determined to a reported 4.3 Å. 3D reconstructions of the 52-nt fluoride riboswitch (blue, PDB-8TJV) and 71-nt Zika virus xrRNA (orange, PDB-8TJQ) were determined using a group I intron scaffold and were reported at 4.46 Å and 5.05 Å respectively. In all these examples, models were built into the determined maps and representative regions of the models fit to density are shown. In these examples, as shown in the zoomed-in insets, the sequence register is uncertain due to the low resolution. The bottom 3D reconstruction of the 86-nt thiamine pyrophosphate riboswitch (grey, PDB-9C6K) was determined in this study using a group II intron as a scaffold to a resolution of 3.1 Å. At this resolution, de novo modeling is possible with the separation between base pairs allowing for the accurate determination of sequence register. In addition, density for the bound thiamine pyrophosphate ligand is visible, which is not possible at the resolution seen in the prior examples.

Figure S2. SHAPE-MaP probing of scaffold-TPP riboswitch construct in the absence and presence of TPP ligand. (A) SHAPE reactivity profiles in the absence or presence of TPP ligand. TPP riboswitch region is emphasized with green box. Riboswitch structural landmarks are highlighted. (B) SHAPE reactivities for the scaffold-riboswitch construct, measured in the presence of TPP, superimposed on a secondary structure model of the complete RNA. (C) Focused view of SHAPE reactivities for the TPP riboswitch aptamer domain, as appended to the scaffold RNA, in the absence and presence of TPP ligand. Secondary structures were modeled using the $\Delta G_{\mathrm{SHAPE}}$ framework (*41*). For the ligand-free state, helices P2alt and P3alt overlap with, but are not identical to, P2 and P3 visualized in the TPP-bound state, consistent with prior studies (*28*). In panels B and C, nucleotides are colored by SHAPE reactivity: red, orange and black correspond to high, medium, and low reactivities, respectively.

Figure S3. Cryo-EM processing workflow of *O.i.*-TPP. (A) Data processing workflow for the *O.i.*-TPP construct focused on *O.i.* and then focused on TPP. This represents our general workflow for the scaffold approach and can be applied to any RNA of interest. **(B)** GSFSC resolution curves and viewing direction distribution plots are shown for the locally refined TPP map. **(C)** GSFSC resolution curves and viewing direction distribution plots are shown for the locally refined *O.i.* map.

Figure S4. Comparison of the scaffold structure with and without an attached RNA. Maps correspond to the scaffold alone and scaffold-TPP samples. The presence of the TPP riboswitch attached to Domain III does not alter the overall 3D reconstruction of the group II intron. A model for the *O.i.* intron (4DS6) was refined in real space into each of the maps and the resulting structure coordinates were aligned using LSQ superpose in COOT (right).

Figure S5. Comparison of cryo-EM and crystal structures of the TPP riboswitch. The cryo-EM derived (cyan) and crystallography derived (2GDI, magenta) structure coordinates were aligned using LSQ superpose in COOT. Full RMSD deviations were determined for the aligned maps in Chimera using Match -> Align. The results of this analysis were graphed (left) and annotated with the corresponding regions of the riboswitch.

Figure S6. Scaffolded *raiA* construct. The RNA sequence for the *O.i.-raiA* construct is shown. Nucleotide changes from the scaffold sequence used for the TPP riboswitch are highlighted in blue. The G to A mutation within the active site is highlighted in red.

Figure S7. Cryo-EM processing workflow of *O.i.-raiA*. (A) Data processing workflow for the *O.i.-raiA* construct is shown. **(B)** Local resolution map of the *raiA* reconstruction. **(C)** Viewing direction distribution plot. **(C)** Gold standard FSC curve.

Figure S8. The P1 helix terminates in a discontinuous GNRA tetraloop. The three-way junction joining the P1, P3, and P4 stems forms a discontinuous GNRA tetraloop. Within the discontinuous GNRA like motif, a trans-S:W base pair between G20:A183 closes the loop. A21, A22, and A183 then form a continuous base stack to complete the motif. The result of this discontinuous tetraloop is an ~180° bend of the helical axis between P1 and P3.

Figure S9. P3 contains an internal loop that forms a 90° helical bend. (A) The I3a-3b internal loop adopts an ~90° bend in the helical axis between the P3a and P3b stems. Both A44 and A46 are extruded out of the internal loop. **(B)** The I1b-1c internal loop contains multiple conserved nucleotides that form a hydrogen bonding network to stabilize the motif. G10 forms a base triple with the G14:C193 cis-W:W base pair.

Figure S10. SHAPE reactivity profile of the *raiA* RNA. **(A)** SHAPE reactivities of the *raiA* RNA as shown. RNA was inserted into the group II intron scaffold. Non-base-paired regions with low SHAPE reactivity are labeled below the nucleotide sequence. **(B)** SHAPE reactivities for the scaffold-*raiA* construct, superimposed on a secondary structure model of the complete RNA as inferred from the cryo-EM structure. Pseudoknot pairings are indicated by dashed lines. Non-base-paired regions with low SHAPE reactivities, indicative of stable, non-canonical structure, are labeled with bold text.

Table S1. Cryo-EM data collection and refinement statistics.

Figure S1.

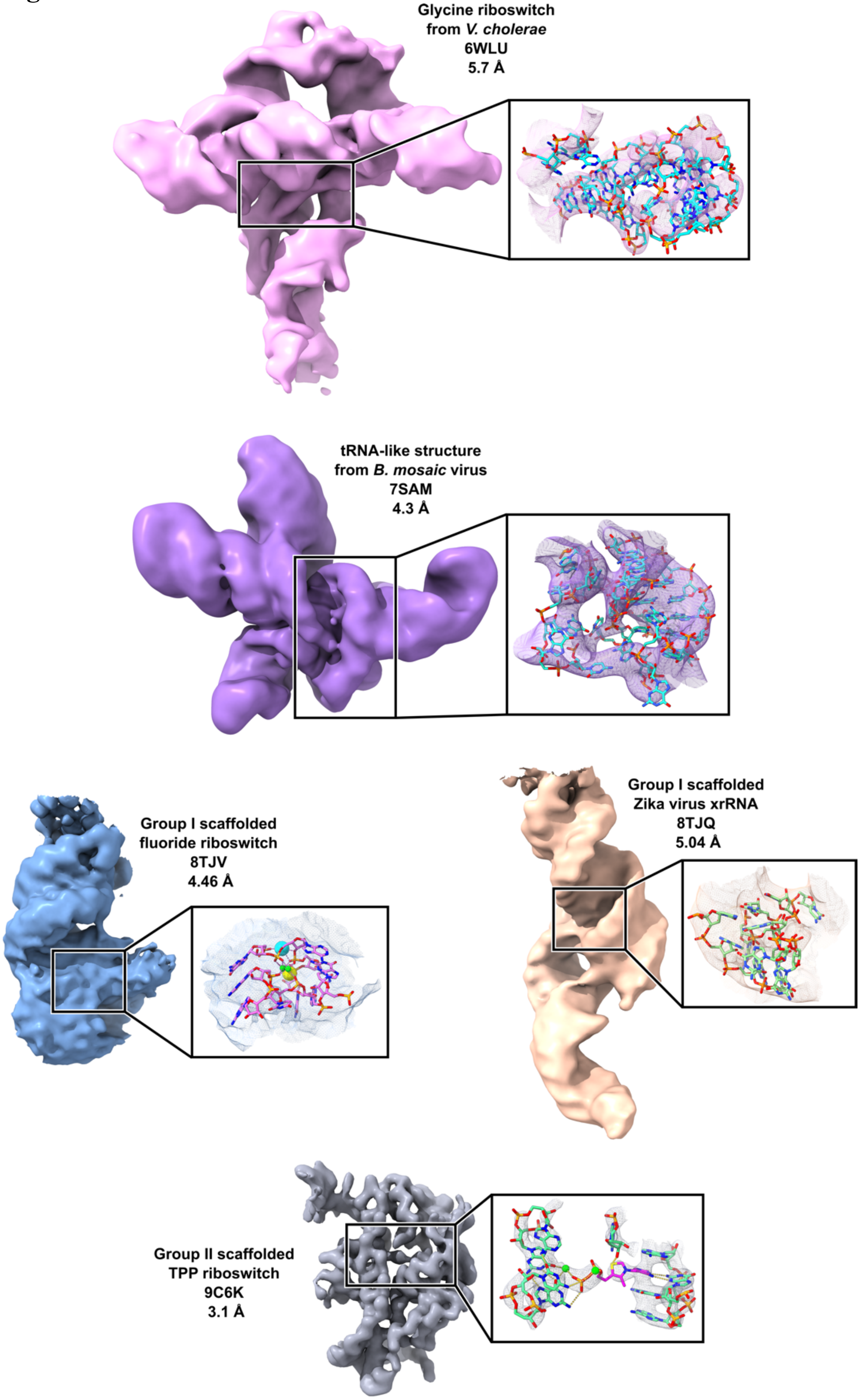
Glycine riboswitch
from *V. cholerae*
6WLU
5.7 Å
tRNA-like structure
from *B. mosaic* virus
7SAM
4.3 Å
Group I scaffolded
fluoride riboswitch
8TJV
4.46 Å
Group I scaffolded
Zika virus xrRNA
8TJQ
5.04 Å
Group II scaffolded
TPP riboswitch
9C6K
3.1 Å

Figure S2

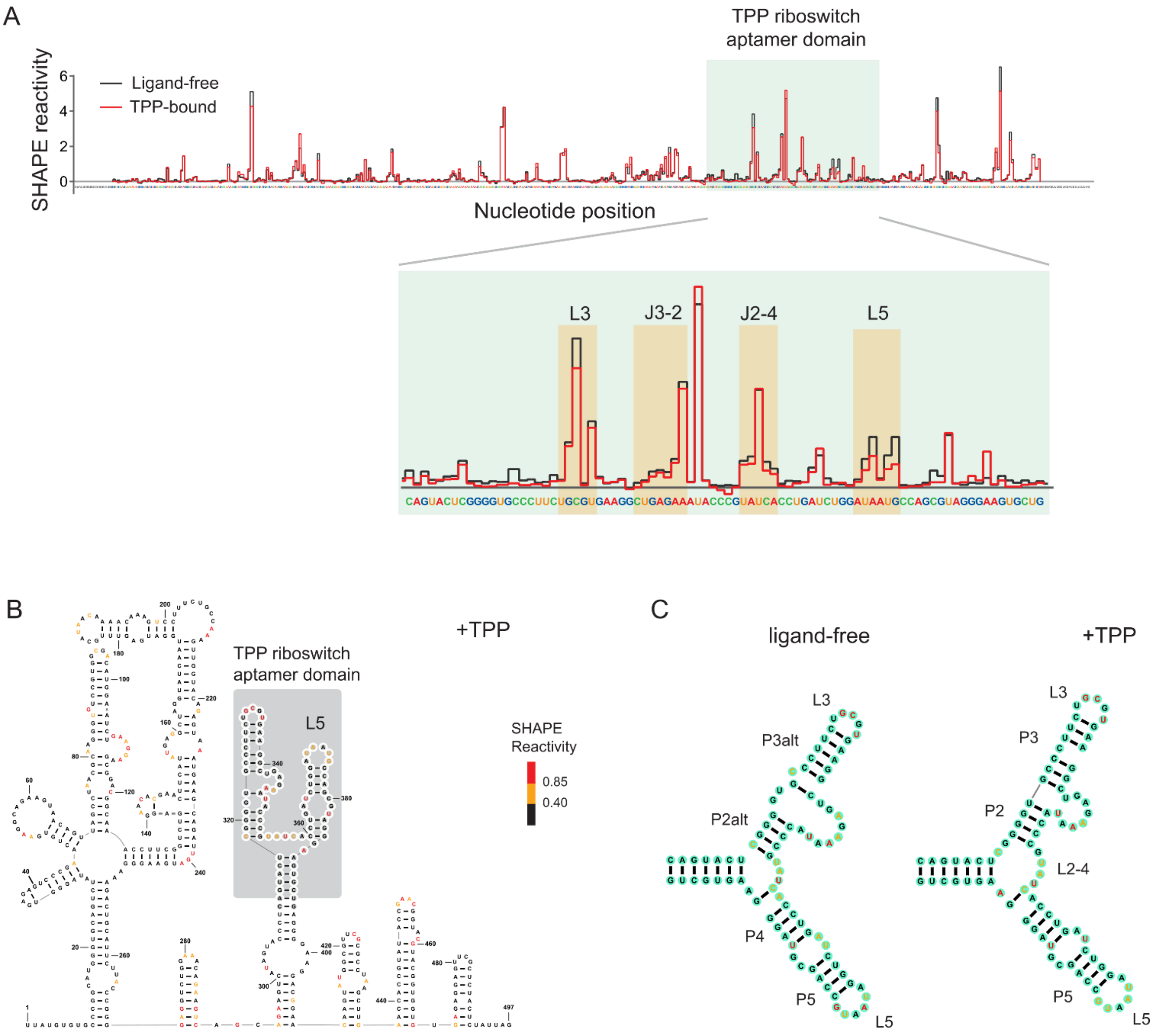

A
TPP riboswitch
aptamer domain
SHAPE reactivity
6
4
2
0
Ligand-free
TPP-bound
Nucleotide position
L3
J3-2
J2-4
L5
B
+TPP
TPP riboswitch
aptamer domain
L5
SHAPE
Reactivity
0.85
0.40
C
ligand-free
+TPP
L3
P3alt
P2alt
P4
P5
L5
L3
P3
P2
L2-4
P5
L5

Figure S3

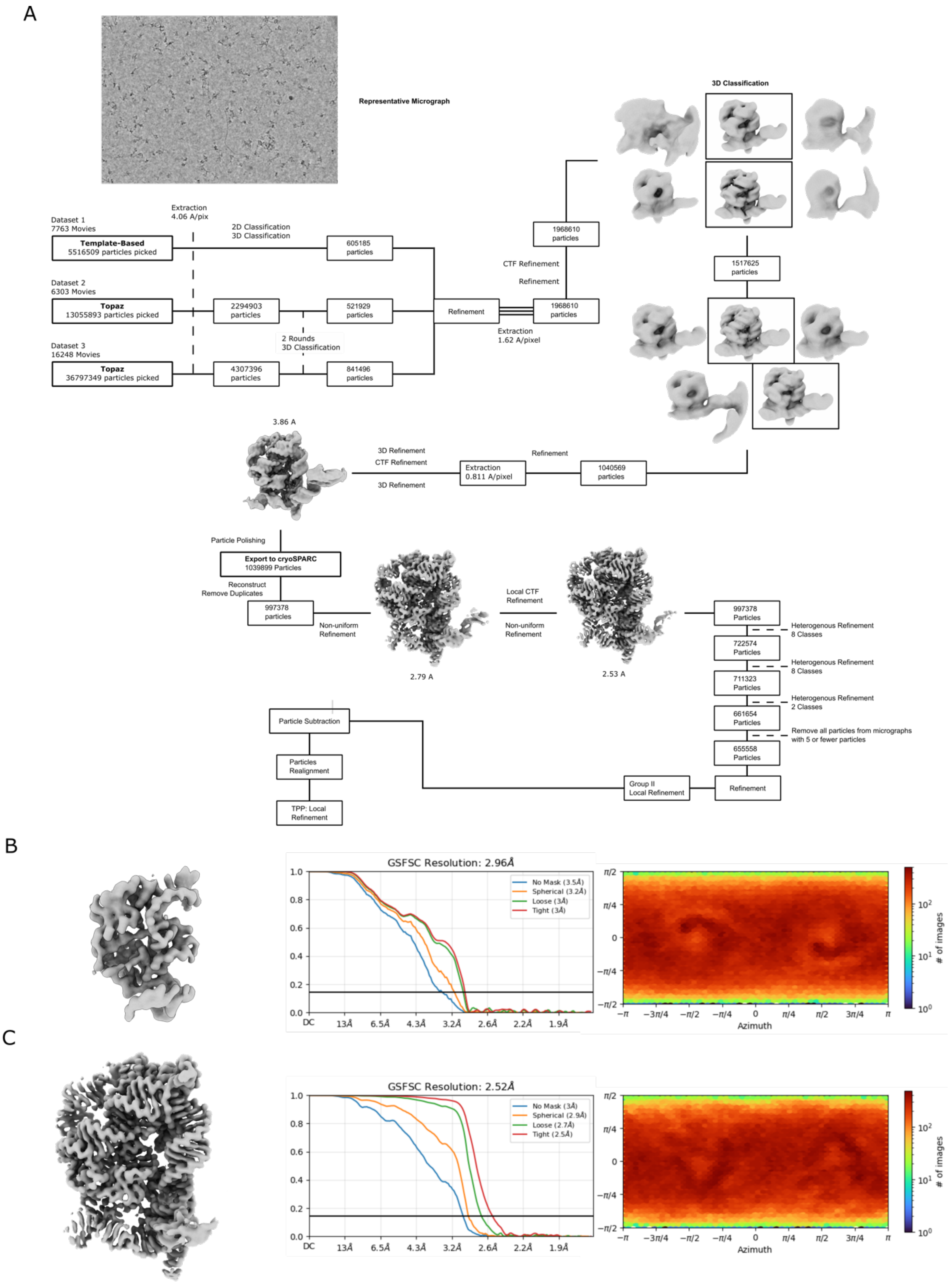
A
Representative Micrograph
3D Classification
Dataset 1
7763 Movies
Template-Based
5516509 particles picked
Extraction
4.06 A/pix
2D Classification
3D Classification
605185
particles
Dataset 2
6303 Movies
Topaz
13055893 particles picked
2294903
particles
521929
particles
Dataset 3
16248 Movies
Topaz
36797349 particles picked
4307396
particles
841496
particles
2 Rounds
3D Classification
Refinement
1968610
particles
CTF Refinement
Refinement
1968610
particles
Extraction
1.62 A/pixel
1517625
particles
3.86 A
3D Refinement
CTF Refinement
3D Refinement
Extraction
0.811 A/pixel
Refinement
1040569
particles
Particle Polishing
Export to cryoSPARC
1039899 Particles
Reconstruct
Remove Duplicates
997378
particles
Non-uniform
Refinement
2.79 A
Local CTF
Refinement
Non-uniform
Refinement
2.53 A
997378
Particles
Heterogenous Refinement
8 Classes
722574
Particles
Heterogenous Refinement
8 Classes
711323
Particles
Heterogenous Refinement
2 Classes
661654
Particles
Remove all particles from micrographs
with 5 or fewer particles
655558
Particles
Refinement
Group II
Local Refinement
Particle Subtraction
Particles
Realignment
TPP: Local
Refinement
B
GSFSC Resolution: 2.96Å
No Mask (3.5Å)
Spherical (3.2Å)
Loose (3Å)
Tight (3Å)
1.0
0.8
0.6
0.4
0.2
0.0
DC
13Å
6.5Å
4.3Å
3.2Å
2.6Å
2.2Å
1.9Å
π/2
π/4
0
−π/4
−π/2
−π
−3π/4
−π/2
−π/4
0
π/4
π/2
3π/4
π
Azimuth
# of images
C
GSFSC Resolution: 2.52Å
No Mask (3Å)
Spherical (2.9Å)
Loose (2.7Å)
Tight (2.5Å)

Figure S4.

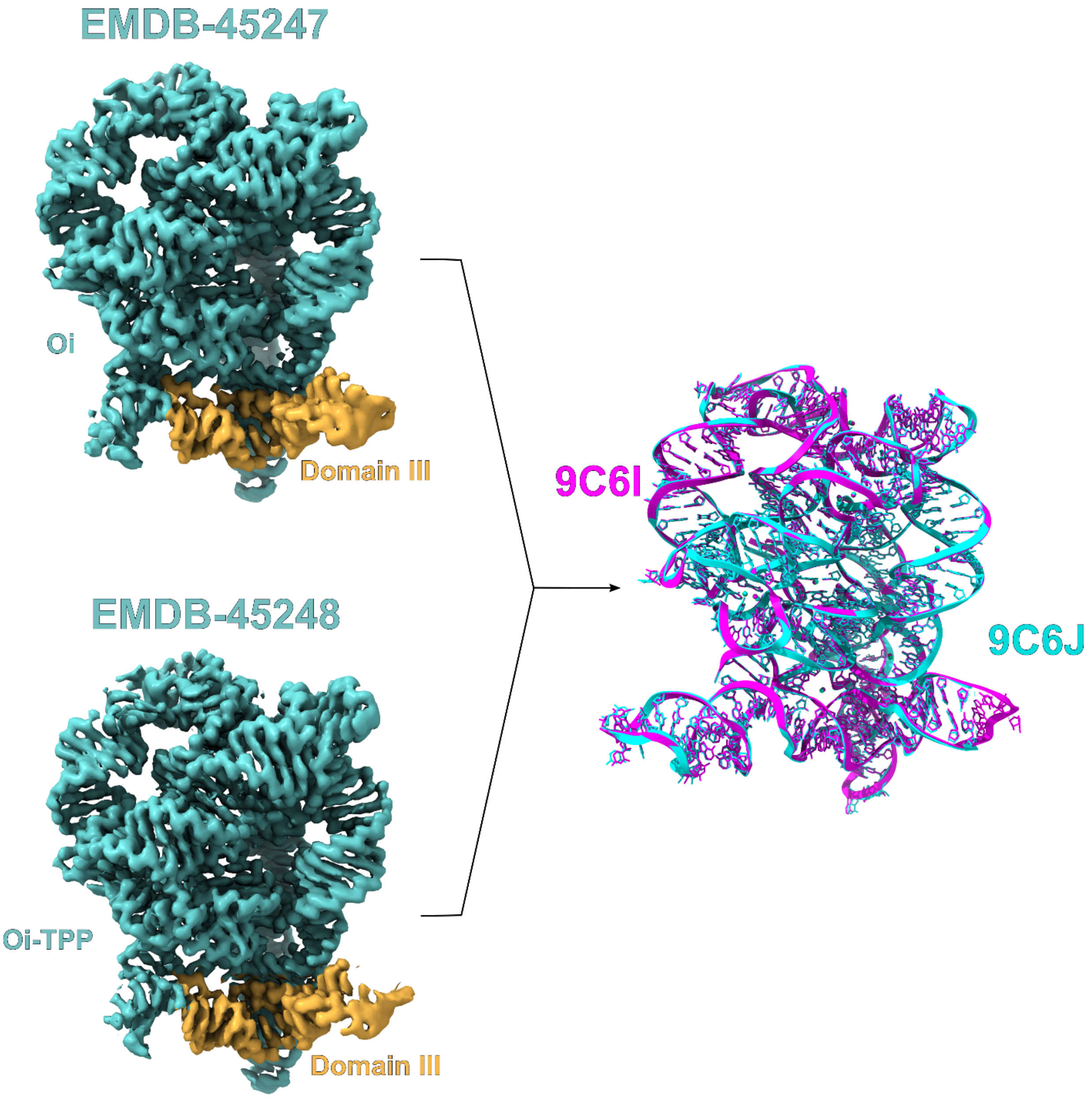
EMDB-45247
Oi
Domain III
EMDB-45248
Oi-TPP
Domain III
9C6I
9C6J

Figure S5.

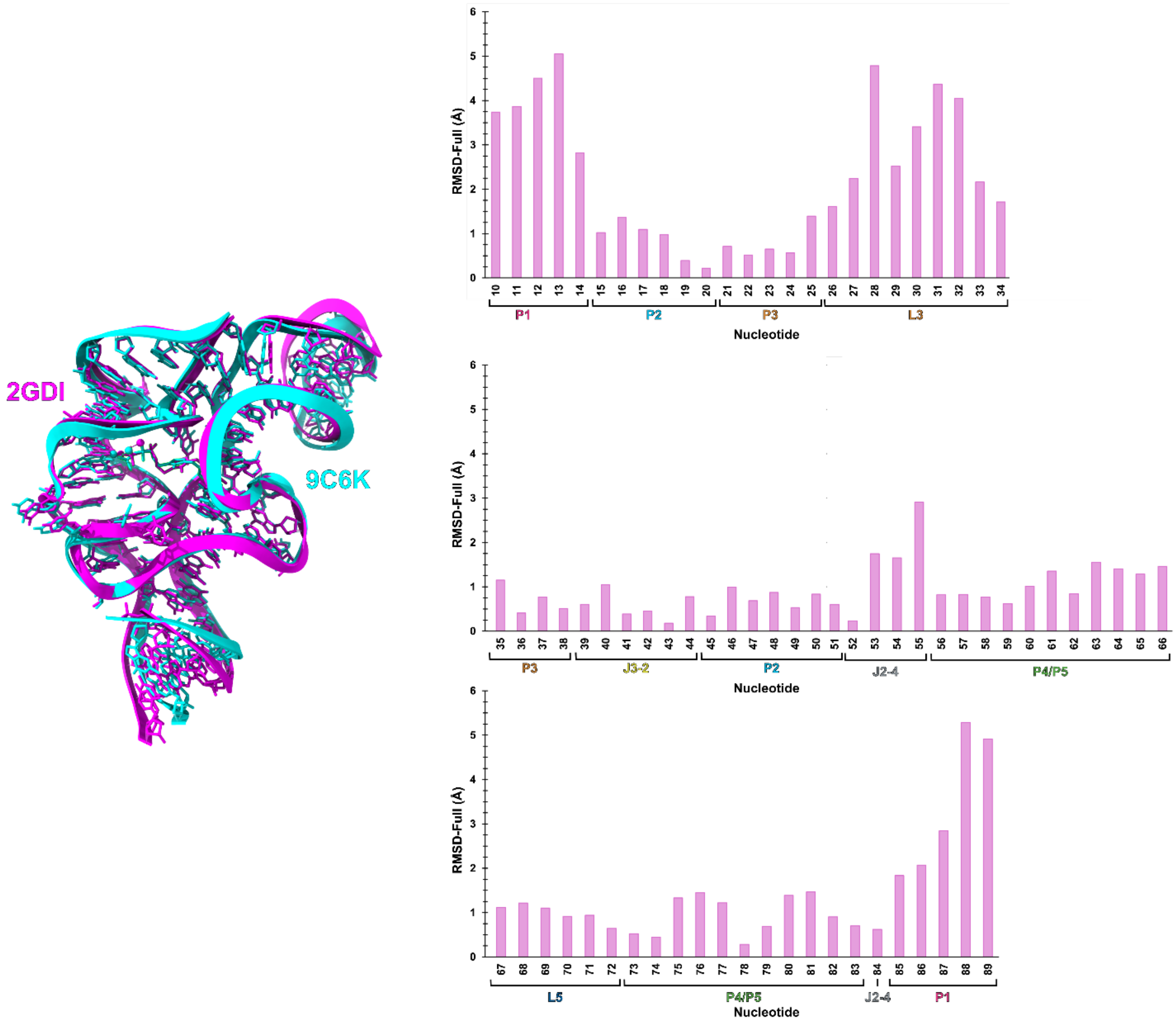
2GDI
9C6K
RMSD-Full (Å)
P1
P2
P3
L3
Nucleotide
RMSD-Full (Å)
P3
J3-2
P2
J2-4
P4/P5
Nucleotide
RMSD-Full (Å)
L5
P4/P5
J2-4
P1
Nucleotide

Figure S6.

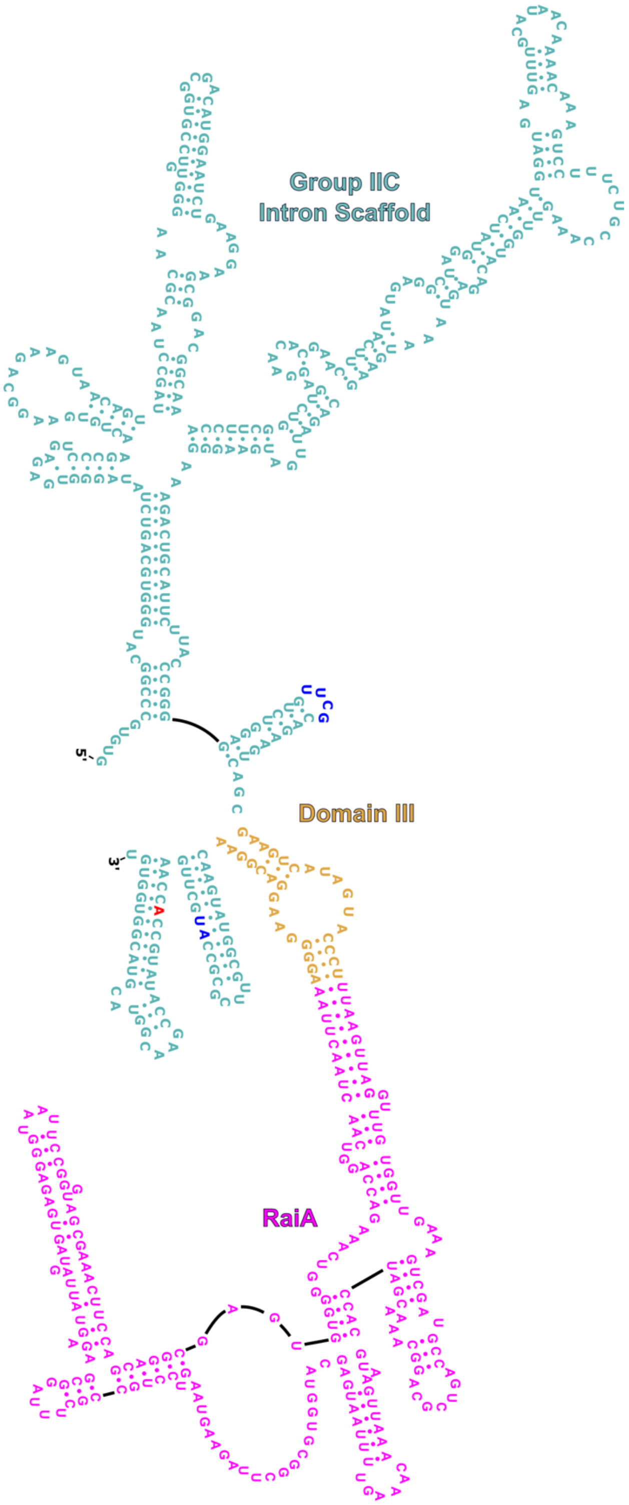

Figure S7.

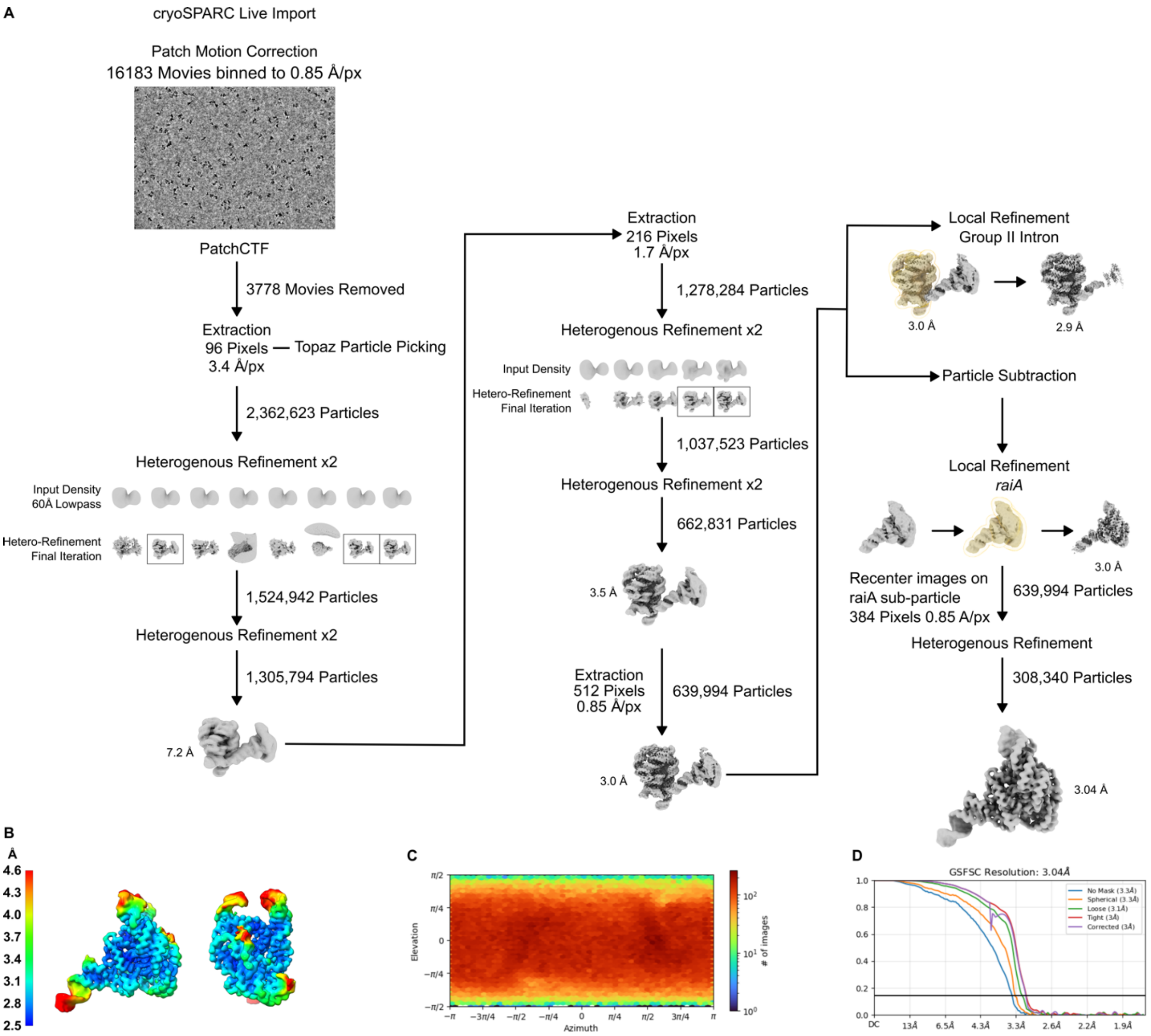
A
cryoSPARC Live Import
Patch Motion Correction
16183 Movies binned to 0.85 Å/px
PatchCTF
3778 Movies Removed
Extraction
96 Pixels
3.4 Å/px
Topaz Particle Picking
2,362,623 Particles
Heterogenous Refinement x2
Input Density
60Å Lowpass
Hetero-Refinement
Final Iteration
1,524,942 Particles
Heterogenous Refinement x2
1,305,794 Particles
7.2 Å
Extraction
216 Pixels
1.7 Å/px
1,278,284 Particles
Heterogenous Refinement x2
Input Density
Hetero-Refinement
Final Iteration
1,037,523 Particles
Heterogenous Refinement x2
662,831 Particles
3.5 Å
Extraction
512 Pixels
0.85 Å/px
639,994 Particles
3.0 Å
Local Refinement
Group II Intron
3.0 Å
2.9 Å
Particle Subtraction
Local Refinement
raiA
Recenter images on
raiA sub-particle
384 Pixels 0.85 A/px
3.0 Å
639,994 Particles
Heterogenous Refinement
308,340 Particles
3.04 Å
B
Å
4.6
4.3
4.0
3.7
3.4
3.1
2.8
2.5
C
Elevation
Azimuth
# of images
D
GSFSC Resolution: 3.04Å
No Mask (3.3Å)
Spherical (3.3Å)
Loose (3.1Å)
Tight (3Å)
Corrected (3Å)
DC
13Å
6.5Å
4.3Å
3.3Å
2.6Å
2.2Å
1.9Å

Figure S8.

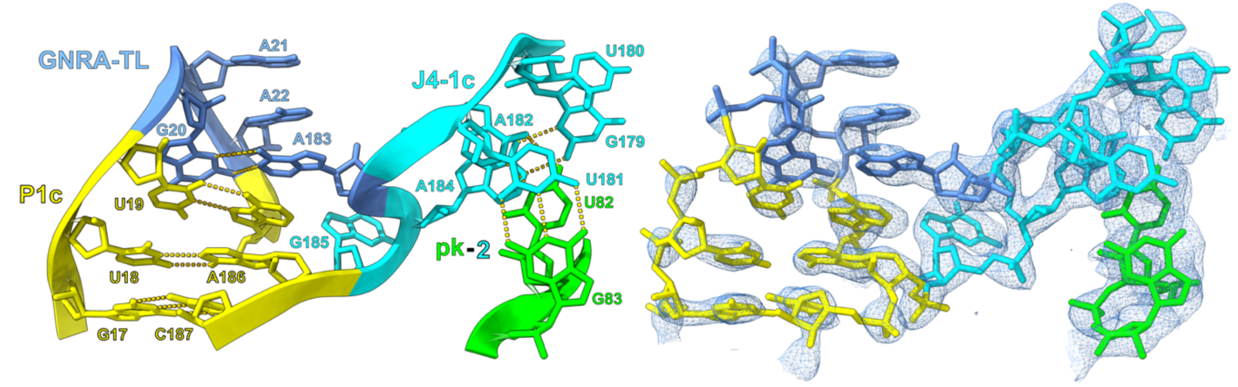
GNRA-TL
A21
A22
G20
A183
P1c
U19
U18
A186
G17
C187
G185
J4-1c
U180
A182
G179
U181
A184
U82
pk-2
G83

Figure S9.

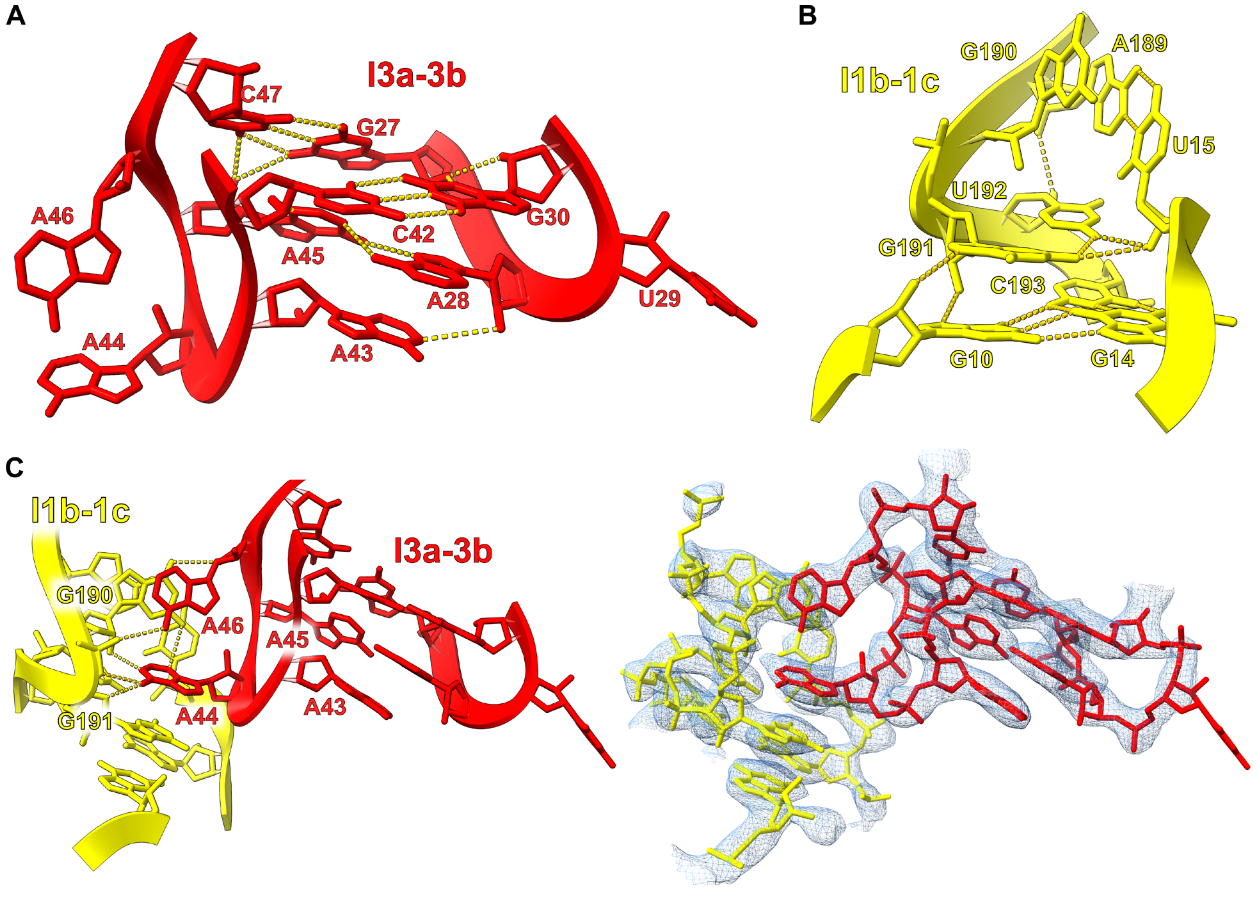
A
I3a-3b
C47
G27
A46
G30
C42
A45
A28
U29
A44
A43
B
G190
A189
I1b-1c
U15
U192
G191
C193
G10
G14
C
I1b-1c
I3a-3b
G190
A46
A45
G191
A44
A43

Figure S10.

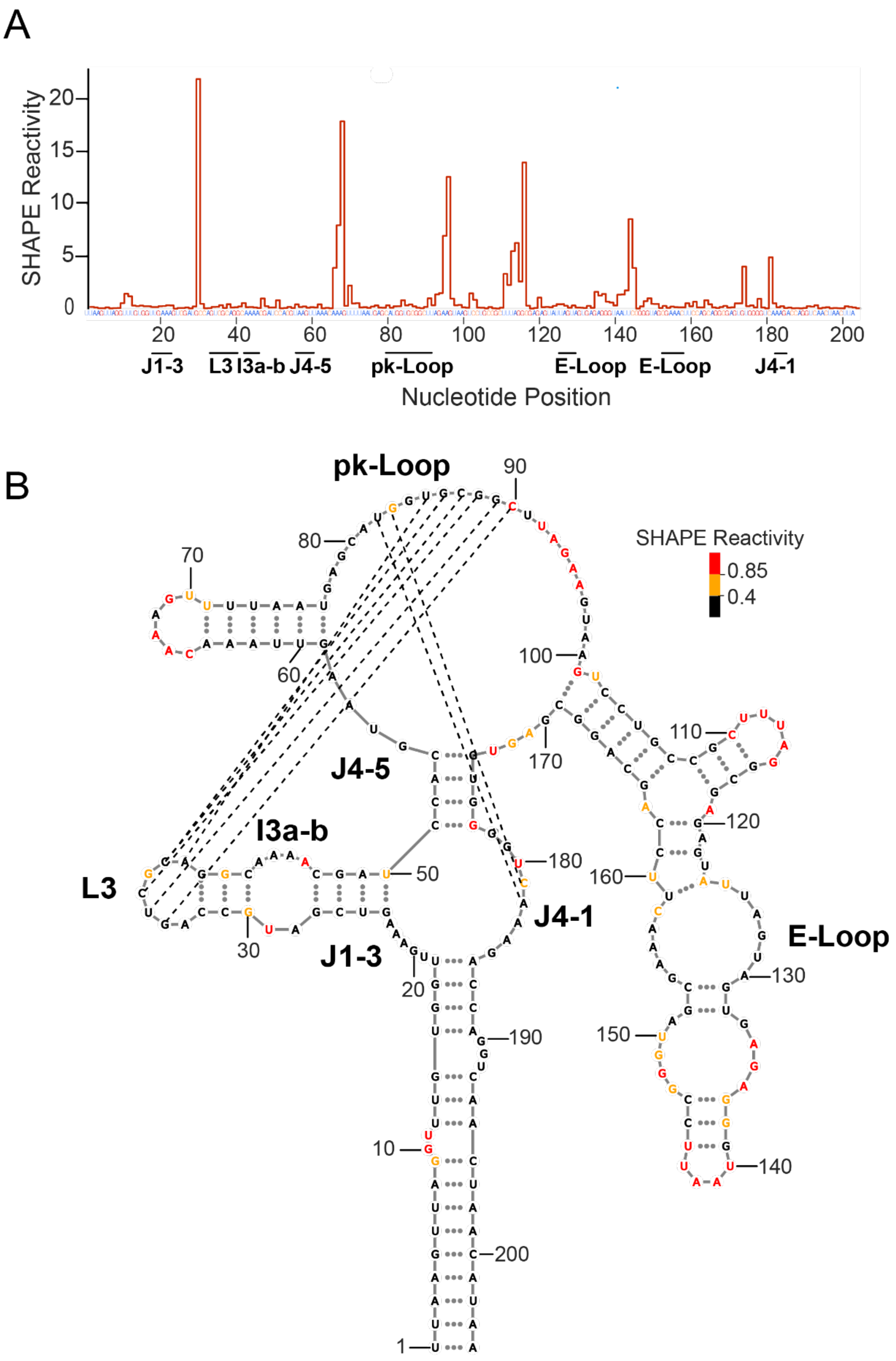
A
SHAPE Reactivity
20
15
10
5
0
20
40
60
80
100
120
140
160
180
200
J1-3
L3
I3a-b
J4-5
pk-Loop
E-Loop
E-Loop
J4-1
Nucleotide Position
B
pk-Loop
SHAPE Reactivity
0.85
0.4
J4-5
I3a-b
L3
J1-3
J4-1
E-Loop

Table S1. Cryo-EM data collection and refinement statistics

	Oi Group II intron	Oi-TPP focused on Oi	Oi-TPP Focused on TPP	Oi-TPP-Apo Focused on TPP	Tel-Oi	O.i.-raiA Focused on *raiA*	
**Data collection**						Full Dataset	Small Dataset
Microscope	FEI Titan Krios	FEI Titan Krios	FEI Titan Krios	FEI Titan Krios	FEI Titan Krios	FEI Titan Krios	FEI Titan Krios
Voltage (kV)	300	300	300	300	300	300	300
Camera	Gatan K3 Summit	Gatan K3 Summit	Gatan K3 Summit	Gatan K3 Summit	Gatan K3 Summit	Gatan K3 Summit	Gatan K3 Summit
Magnification	105,000	105,000	105,000	105,000	105,000	105,000	105,000
Nominal defocus range (µm)	-0.5/-1.5	-0.5/-1.5	-0.5/-1.5	-0.5/-1.5	-0.8/-1.9	-0.8/-2	-0.8/-2
Exposure time (s)	4.46	4.46	4.46	4.46	5.43	4.5	4.5
Number of frames	50	50	50	50	80	50	50
Dose rate (e^-/Pixel/s)	8	8	8	8	8.5	8	8
Total dose (e^-/Å^2)	50	50	50	50	58	50	50
Pixel size (Å)	0.811	0.811	0.811	0.811	0.811	0.84	0.84
Micrographs collected	5,724	30,314	30,314	15,876	2,596	16,183	1,328
Micrographs processed	5,381	26,954	26,954	11,485	1,775	12,405	1,194
Total particles	1,645,938	1,968,610	1,968,610	1,032,440	331,439	2,363,623	263,878
Particles used in final map	382,753	655,558	655,558	106,248	34,822	308,340	34,517
Map resolution global (FSC 0.143) masked	2.56	2.41	2.94	4.73	4.70	3.04	3.78
**Model composition**							
Non-hydrogen atoms	8471	8471	1740			4318	
Protein residues	0	0	0			0	
RNA bases	393	393	80			200	
Ligands	21	21	3			22	
**Refinement (PHENIX)**							
Refinement package		**Real space refinement**				**Real space refinement**	
CC (volume)	0.89	0.87	0.84			0.90	
CC (mask)	0.89	0.87	0.84			0.90	
CC (peak)	0.86	0.75	0.76			0.86	
FCS map-to-model (0.5) masked	2.80	2.86	3.07			3.12	
Map sharpening B-factor (Å^2)	-97.9	-86.4	-118.8			-112.0	
**Rms deviations**							
Bond length (Å)	0.008	0.008	0.007			0.006	
Bond angles (°)	0.667	0.786	0.756			0.68	
**Validation**							
Molprobity score	2.72	2.84	2.90			2.58	
All-atom clashscore	11.33	15.41	17.91			7.88	
